# A Fiber Bragg Grating Sensing Based Triaxial Vibration Sensor

**DOI:** 10.3390/s150924214

**Published:** 2015-09-18

**Authors:** Tianliang Li, Yuegang Tan, Yi Liu, Yongzhi Qu, Mingyao Liu, Zude Zhou

**Affiliations:** School of Mechanical and Electronic Engineering, Wuhan University of Technology, Wuhan 430070, China; E-Mails: tianliangliwhut@sina.com (T.L.); wgdliuyi2001@126.com (Y.L.); quyongzhi@163.com (Y.Q.); lmylyf@126.com (M.L.); zudezhou@whut.edu.cn. (Z.Z.)

**Keywords:** fiber Bragg grating, triaxial, vibration sensor

## Abstract

A fiber Bragg grating (FBG) sensing based triaxial vibration sensor has been presented in this paper. The optical fiber is directly employed as elastomer, and the triaxial vibration of a measured body can be obtained by two pairs of FBGs. A model of a triaxial vibration sensor as well as decoupling principles of triaxial vibration and experimental analyses are proposed. Experimental results show that: sensitivities of 86.9 pm/g, 971.8 pm/g and 154.7 pm/g for each orthogonal sensitive direction with linearity are separately 3.64%, 1.50% and 3.01%. The flat frequency ranges reside in 20–200 Hz, 3–20 Hz and 4–50 Hz, respectively; in addition, the resonant frequencies are separately 700 Hz, 40 Hz and 110 Hz in the *x*/*y*/*z* direction. When the sensor is excited in a single direction vibration, the outputs of sensor in the other two directions are consistent with the outputs in the non-working state. Therefore, it is effectively demonstrated that it can be used for three-dimensional vibration measurement.

## 1. Introduction

Vibration monitoring is a hot issue in mechanical engineering. Mechanical vibrations often cause mechanical equipment damage or failure. Therefore, vibration sensors are considered the key technology in monitoring mechanical equipment. Conventional electronic sensors are often used for vibration measurements, but they have some drawbacks, such as susceptibility to electromagnetic interference, difficulty to make a large number of sensors multiplex and so on.

Compared with traditional electronic sensors, fiber Bragg grating (FBG) sensors are resistant to electromagnetic interference and high temperature, small in size, light in weight and easy for conducting distributed dynamic measurement [1,2]. Many FBG based vibration sensors have been reported. Todd *et al.* presented a FBG accelerometer based on a flexural beam. Sensitivity of the sensor is 212.5 pm/g with resonant frequency up to 110 Hz [3]. An *L*-shaped modified cantilever beam shaped FBG based accelerometer with self temperature compensation in reference [4], which sensitivity is 46 pm/g for frequency below 50 Hz and 306 pm/g for frequency above 150 Hz. Au *et al.* proposed a tapered plate FBG accelerometer, its sensitivity is 18.93 με/g, and maximum input signal frequency up to 150 Hz [5]. Basumallick *et al.* presented a method to improve the sensitivity of sensor by altering the distance between the axis of the FBG sensor to the neutral axis of the cantilever, it is demonstrated that a sensitivity about 1062 pm/g can be achieved, the enhancement almost by a factor of three as compared to that of the conventional cantilever-mass FBG accelerometer of similar bandwidth [6]. Liu *et al.* proposed an FBG accelerometer based on double diaphragm, the flat frequency range is from 50 to 800 Hz with corresponding sensitivity range from 23.8 to 45.9 pm/g, and cross-axis sensitivity is less than 2.1% of the main-axis sensitivity [7]. In addition, Li *et al.* designed an FBG vibration sensor by a diaphragm to measure the vibration of shaft [8]. The above sensors are mainly used to measure the single vibration; they can’t employ the multi-dimensional vibration measurement.

Therefore, some researchers have proposed multi-dimensional vibration sensors. Munendhar *et al.* presented a design strategy of an FBG-based all-optical vibration sensor. Vibration of sensors displace the seismic mass fixed between two flexural beams, thus inducing a variable strain in the two beams. A maximum peer-to-peer wavelength shift in this case is 1.48 nm under input signal’s frequency ranging from 25 Hz to 100 Hz [9]. Antunes *et al.* proposed biaxial optical accelerometer with temperature and cross-axis insensitivity. It is based on four FBGs placed in opposite positions. Sensitivities of 87.848 and 92.351 pm/g for each sensitive direction with resonant frequencies of 846.01 and 845.33 Hz, respectively [10]. Morikawa *et al.* proposed a triaxial Bragg grating accelerometer. The optical fibers with FBGs are used as spring elements, temperature effects are compensated by having two sensors in the same fiber, and the triaxial construction reduces transverse sensitivity [11]. Abushagur *et al.* used the same principle as the literature [11] to design three-axes fiber Bragg grating accelerometer [12]. On the basis of literature [11,12] principle, Jiang *et al.* presented the design, simulation and calibration of the three-axis accelerometer. The fully described dynamic sensitivity of three-axis accelerometers represented by a three-by-three matrix is given. The experiment results indicate that the accelerometer has a sensitivity of 0.068 V/g in a measured full scale of ±2.5 m/s^2^ [13]. The essential principle of the triaxial FBG vibration in literature [10,11,12,13] is the same, so they have common drawbacks, such as the size is larger, it is not easy to encapsulate, and so on.

Based on the above mentioned drawbacks of an FBG based sensor, a fiber Bragg grating sensing based triaxial vibration sensor has been presented in this paper. The triaxial vibration of measured body can be decoupled, attained by two pairs of FBGs which are horizontal and vertical arranged on the base of sensor. The model of the sensor as well as decoupling principle for triaxial vibration measurement and experimental analyses are introduced in the next section. The remaining paper mainly falls into the following parts: principle and model of sensors, and experimental analysis of sensing properties of sensors.

## 2. Model and Principles of a Triaxial Vibration Sensor

**Figure 1:**
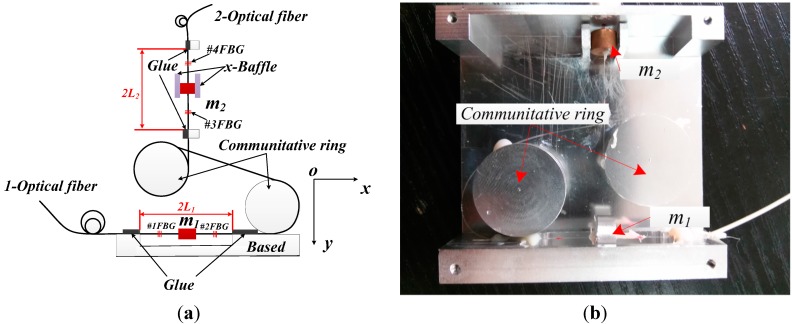
Schematic diagram and photograph of an FBG based triaxial vibration sensor.

Figure 1 is the schematic diagram and photograph of an FBG based triaxial vibration sensor. In the horizontal direction, the *m*_1_ is fixed on the 1-optical fiber, and the *z* direction freedom of the *m*_1_ is limited by the Base. The #1FBG and #2FBG are placed on both sides of *m*_1_ in the one-optical fiber. At first, fixing one end of the optical fiber with glue; and then exerting a certain tension on the other end of optical fiber; finally using glue fixed on the other end. The vibration can be decoupled by the double FBGs’ center wavelength with subtraction/addition in the *x/y* direction. On the vertical direction, the *m*_2_ and #3 and #4 FBG are fixed by the same method of *m*_1_. The *x*-Baffle is used to limit the *x* direction freedom of the *m*_2_. In order to ensure that the sensor can be measured the triaxial vibration, which is in the same place of the measured body. Thus, block *m*_2_ and *m*_1_ are fixed in the same vertical line. The *z*-direction (*z*-direction is perpendicular to the *xoy* plane) vibration can be attained by addition of the #3FBG and #4FBG center wavelength shift. The #1FBG/#2FBG and #3FBG/#4FBG are connected by an optical fiber; in addition, the optical fiber is enwound on the communitative ring, which is used to change the direction of the optical fiber.

### 2.1. Axial Vibration of Optical Fiber

**Figure 2:**
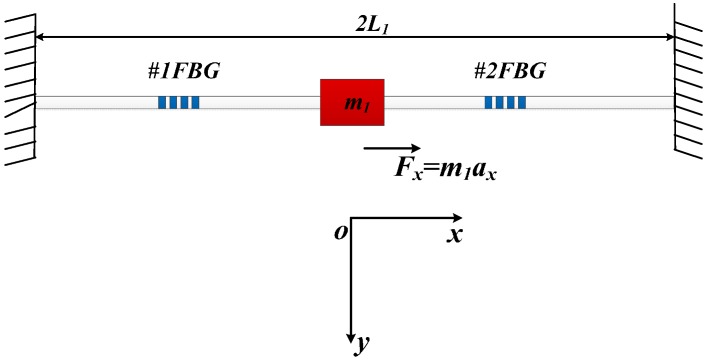
Vibration model of optical fiber in axial direction.

From Figure 1, the vibration principle of FBGs for measuring the vibration is the same in the horizontal and vertical direction. Thus, we select one-optical fiber as the research object, which is the horizontally arranged in the sensor (Figure 2). When the *x*-direction inertial force *F_x_* = *m*_1_*a_x_* is applied on the block *m*_1_, the left and right part of optical fiber will be stretched and compressed. The stretched and compressed displacements are separately signed as *x* and –*x*. According to the mechanics of materials, the optical fiber axial stiffness *K_x_* can be expressed as:

(1) $$K_{x}=\frac{2E_{f}A_{f}}{L_{1}}$$

where *E_f_* is Young’s Modulus of optical fiber, *A_f_* means cross-sectional area of the optic fiber. *L_1_* is the effective length of horizontally arranged optical fiber.

According to the theory of the vibration, when the frequency of vibration *w* << *w_x_*, the displacement *x* can be expressed as:

(2) $$x=\frac{a_{x}}{{w_{x}}^{2}}=\frac{m_{1}a_{x}}{K_{x}}=\frac{m_{1}a_{x}L_{1}}{2E_{f}A_{f}}$$

The *w_x_* is resonant frequency of optical fiber in the horizontal direction. From Figure 1, we can easily get that the #1FBG will be compressed or stretched under the inertial force, and its strain is equal to *ε_x_*. Due to the #2 FBG suffers to opposite stress, so its strain is −*ε_x_*. Combing the Equation (2), the *ε_x_* can be written as:

(3) $$\varepsilon_{x}=\frac{x}{L}=\frac{m_{1}L_{1}}{2E_{f}A_{f}}a_{x}=S_{x}a_{x}$$

where *S_x_* is the sensitivity of acceleration *a_x_* along the *x*-vibration direction.

### 2.2. Transverse Vibration of Optical Fiber

**Figure 3:**
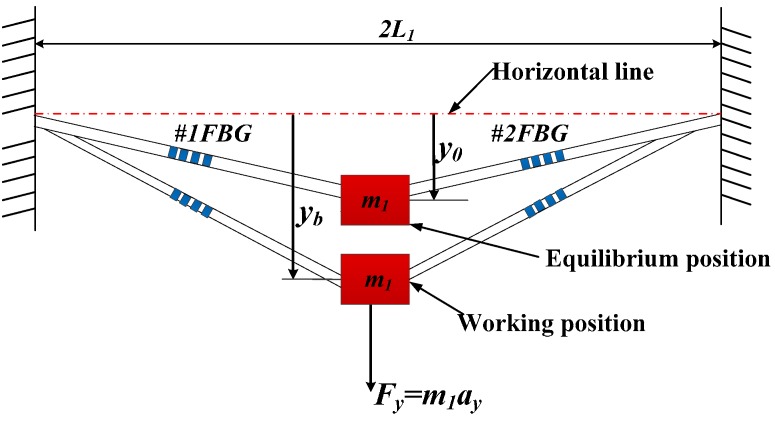
Vibration model of optical fiber in transverse direction.

Figure 3 is the vibration model of optical fiber in transverse direction. There are two kinds of states of block *m*_2_—when the *a_y_* = 0, *y*-direction inertial force *F_y_* = *m*_1_*a_y_* = 0, and the block *m*_1_ is in equilibrium position. The distance *y*_0_ is between the equilibrium position of *m*_1_ and the horizontal line, which is through the two fixed point of optical fiber. When *a_y_* ≠ 0, the block *m*_1_ is in working position under *y*-direction inertial force *F_y_*. The mark *y_b_* represents the distance between the working position and the horizontal line. Thus, when the block *m*_1_ moves from equilibrium position to working position, the optical fiber’s strain increment *ε_y_* can be expressed as:

(4) $$\varepsilon_{y}=\frac{\sqrt{L_{1}^{2}+y^{2}}-\sqrt{L_{1}^{2}+y_{b}^{2}}}{L_{1}}$$

According to Taylor formula, Equation (4) can be rewritten as:

(5) $$\varepsilon_{y}=\frac{L_{1}+\frac{y^{2}}{2L_{1}}+ο(y^{3})-(L_{1}+\frac{y_{b}^{2}}{2L_{1}}+ο(y_{0}^{3}))}{L_{1}}\approx \frac{\left(y-y_{b})(y+y_{b}\right)}{2L_{1}^{2}}$$

Block *m*_1_ has a small vibration (up and down) around the equilibrium position, so there exists the *y* − *y_0_* << *y_0_*. Equation (5) can be simplified as:

(6) $$\varepsilon_{y}=\frac{\left(y-y_{b})(y-y_{b}+2y_{b}\right)}{2L_{1}^{2}}\approx \frac{(y-y_{b})2y_{b}}{2L_{1}^{2}}$$

There exists an initial strain *ε_y_*_0_ of 1-optical fiber under axial prestress in the process of sensor encapsulation. When the block *m*_1_ is in the equilibrium position, there exists an increment of *ε_yb_* compared to prestressed 1-optical fiber in the horizontal direction, which can be expressed as:

(7) $$\varepsilon_{yb}=\frac{\sqrt{L_{1}^{2}+y_{b}^{2}}-L_{1}}{L_{1}}$$

According to the statics and vibration mechanics, the horizontal resonant frequency *w_y_* of the optical fiber can be written as [14]:

(8) $$w_{y}=\sqrt{\frac{2E_{f}A_{f}(\varepsilon_{y0}+\varepsilon_{yb})}{(\varepsilon_{yb}+1)L_{1}m_{1}}}$$

Using the similar method of Equation (2), the increment *y* − *y_b_* can be expressed as:

(9) $$y-y_{b}=\frac{a_{y}}{w_{y}^{2}}$$

Combing Equations (7), (8) with (9), the relationship between *ε_y_* and *a_y_* can be written as:

(10) $$\varepsilon_{y}=\frac{m_{1}(\varepsilon_{yb}+1)\sqrt{\varepsilon_{yb}^{2}+2\varepsilon_{yb}}}{2E_{f}A_{f}(\varepsilon_{y0}+\varepsilon_{yb})}a_{y}=S_{y}a_{y}$$

where *S_y_* is sensitivity of acceleration *a_y_* along *y*-vibration direction.

### 2.3. Decoupling Principle of Triaxial Vibration Sensor

Assuming the working environment temperature of sensors is constant, according to the principle of FBG sensing, the center wavelength shifts of each FBG can be expressed as:

(11) $$\left(\begin{array}{c} \Delta \lambda_{1}/\lambda_{1}\\ \Delta \lambda_{2}/\lambda_{2}\\ \Delta \lambda_{3}/\lambda_{3}\\ \Delta \lambda_{4}/\lambda_{4} \end{array}\right)=(1-\rho_{e})\left(\begin{array}{c} \Delta \varepsilon_{1}\\ \Delta \varepsilon_{2}\\ \Delta \varepsilon_{3}\\ \Delta \varepsilon_{4} \end{array}\right)$$

where Δ*λ*_1_, Δ*λ*_2_, Δ*λ*_3_ and Δ*λ*_4_ are the center wavelength shift of #1FBG, #2FBG, #3FBG and #4FBG, respectively; *λ*_1_, *λ*_2_, *λ*_3_ and *λ*_4_ separately represent the center wavelength of #1FBG, #2FBG, #3FBG and #4FBG; *ρ_e_* represents the strain-optic coefficient of optical fiber; Δ*ε*_1_, Δ*ε*_2_, Δ*ε*_3_ and Δ*ε*_4_ are the axial strain of #1FBG, #2FBG, #3FBG and #4FBG, respectively. Thus from Equation (11), the strain *ε_y_*_0_ and *ε_yb_* can be measured by the center wavelength shift of FBGs.

According to Equations (3) and (10), we can find that both of them can be treated as linear system for the acceleration, so the outputs of linear system can be added together for simultaneous vibration along the *x* and *y* direction. When block *m*_1_ is exerted on inertial force *F_xy_* = *m*_1_*a_xy_* (*a_xy_* can be decomposed into *a_x_* and *a_y_*), the *Δλ_1_* and *Δλ_2_* can be expressed as:

(12) $$\begin{array}{l} \left(\begin{array}{c} \Delta \lambda_{1}/\lambda_{1}\\ \Delta \lambda_{2}/\lambda_{2} \end{array}\right)=(1-P_{e})\left(\begin{array}{cc} 1 & 1\\ -1 & 1 \end{array}\right)\left(\begin{array}{c} \varepsilon_{x}\\ \varepsilon_{y} \end{array}\right)\\ =(1-P_{e})\left(\begin{array}{cc} \frac{m_{1}L_{1}}{2E_{f}A_{f}} & \frac{m_{1}(\varepsilon_{yb}+1)\sqrt{\varepsilon_{yb}^{2}+2\varepsilon_{yb}}}{2E_{f}A_{f}(\varepsilon_{y0}+\varepsilon_{yb})}\\ -\frac{m_{1}L_{1}}{2E_{f}A_{f}} & \frac{m_{1}(\varepsilon_{yb}+1)\sqrt{\varepsilon_{yb}^{2}+2\varepsilon_{yb}}}{2E_{f}A_{f}(\varepsilon_{y0}+\varepsilon_{yb})} \end{array}\right)\left(\begin{array}{c} a_{x}\\ a_{y} \end{array}\right)=(1-P_{e})\left(\begin{array}{cc} S_{x} & S_{y}\\ -S_{x} & S_{y} \end{array}\right)\left(\begin{array}{c} a_{x}\\ a_{y} \end{array}\right) \end{array}$$

Using the similar method to study on the relationship between Δ*λ*_3_/Δ*λ*_4_ and *a_y_*/*a_z_*, it can be expressed as:

(13) $$\left(\begin{array}{c} \Delta \lambda_{3}/\lambda_{3}\\ \Delta \lambda_{4}/\lambda_{4} \end{array}\right)=(1-P_{e})\left(\begin{array}{cc} S_{y1} & S_{z}\\ -S_{y1} & S_{z} \end{array}\right)\left(\begin{array}{c} a_{y}\\ a_{z} \end{array}\right)$$

where *S_y_*_1_ means response sensitivity of #3FBG and #4FBG for the *y*-vibration direction, *S_z_* is sensitivity of acceleration *a_z_* along *z*-vibration direction.

According to Equation (13), the expression of *a_z_* can be simplified as:

(14) $$\frac{\Delta \lambda_{3}}{\lambda_{3}}+\frac{\Delta \lambda_{4}}{\lambda_{4}}=2S_{z}a_{z}=\frac{m_{2}(\varepsilon_{zb}+1)\sqrt{\varepsilon_{zb}^{2}+2\varepsilon_{zb}}}{E_{f}A_{f}(\varepsilon_{z0}+\varepsilon_{zb})}a_{z}$$

where strain *ε_z_*_0_ of two-optical fiber under axial prestress in the process of sensor encapsulation; *ε_yb_* means the strain increment compared to prestressed two-optical fiber.

Combining Equation (12) with Equation (13), the triaxial vibration measurement matrix of sensor can be attained by the center wavelength shift of FBGs. It can be written as:

(15) $$\left(\begin{array}{cccc} \Delta \lambda_{1} & -\Delta \lambda_{2} & 0 & 0\\ \Delta \lambda_{1} & \Delta \lambda_{2} & 0 & 0\\ 0 & 0 & \Delta \lambda_{3} & \Delta \lambda_{4} \end{array}\right)\left(\begin{array}{c} 1/\lambda_{1}\\ 1/\lambda_{2}\\ 1/\lambda_{3}\\ 1/\lambda_{4} \end{array}\right)=\left(\begin{array}{ccc} 2S_{x} & 0 & 0\\ 0 & 2S_{y} & 0\\ 0 & 0 & 2S_{z} \end{array}\right)\left(\begin{array}{c} a_{x}\\ a_{y}\\ a_{z} \end{array}\right)$$

Based on Equation (15), the acceleration *a_x_*, *a_y_* and *a_z_* can be easily achieved by the FBGs’ center wavelength shift. Thus, it can be used to measure the triaxial vibration of measured body.

## 3. Experiments of Sensing Characteristics and Discussions

**Figure 4:**
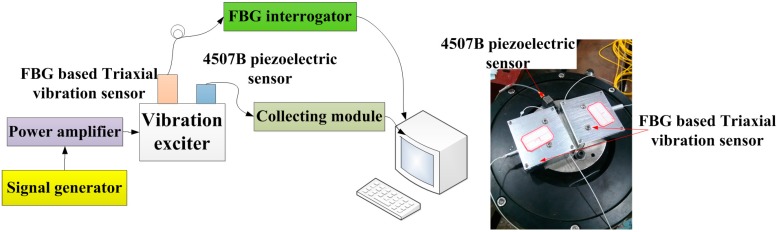
Schematic diagram and photograph of sensing characteristics experiment.

Figure 4 shows schematic diagram and photograph of sensing characteristics experiment for the triaxial vibration sensor. The FBG vibration sensor and 4507B piezoelectric sensor (sensitivity: 9.91 mV/s^2^) are fixed on the vibration exciter. In order to realize calibration of the FBG vibration sensor, we used the 4507B piezoelectric sensor as the standard reference. The driving signal of vibration exciter is handled by the signal generator and power amplifier. The center wavelength signal of FBG and 4507B piezoelectric sensor’s signal can be collected by the FBG interrogator (sample rate: 4K, resolution ratio: 0.1 pm) and collecting module, respectively. Each of the FBG center wavelengths of the triaxial vibration sensor are shown in Table 1.

**Table 1:** Each of the FBG center wavelengths of the triaxial vibration sensor.

Number of FBGs	#1FBG	#2FBG	#3FBG	#4FBG
Initial center wavelength/nm	1538.047	1543.369	1551.967	1557.016
Center wavelength after prestress/nm	1538.715	1544.052	1552.312	1557.407
Center wavelength shift after prestress/nm	0.668	0.683	0.345	0.391

### 3.1. Dynamic Characteristic Experiments of Sensor

**Figure 5:**
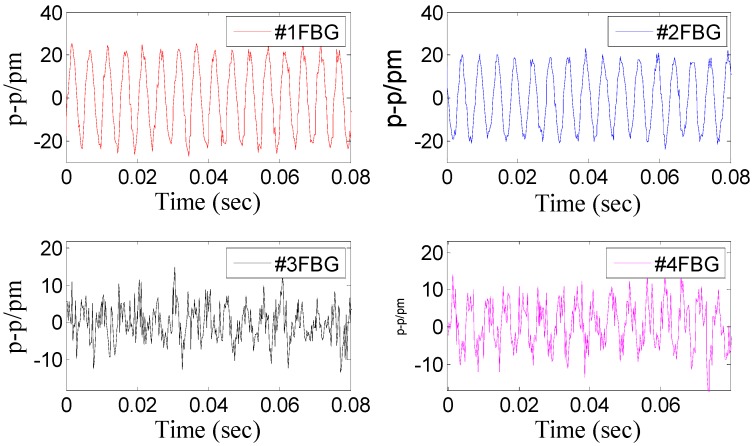
Time domain signal of each FBG under *x*-direction excitation with the acceleration—10 m/s^2^-200 Hz.

**Figure 6:**
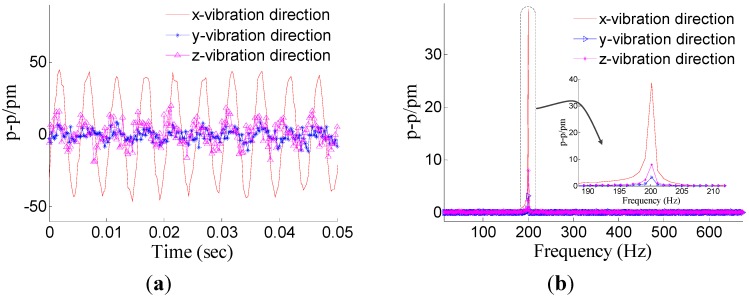
Response of the three directions under *x*-direction excitation with the acceleration—10 m/s^2^-200 Hz. (**a**) Time domain response map. (**b**) Spectrum map.

Dynamic property is one of the important indicators to evaluate performance of sensor. In order to investigate the dynamic properties, the sensor is fixed on the vibration exciter (Figure 4), and the *x*-direction is treated as the main vibration direction. During the experiment, the frequency of acceleration changes within 20 to 1000 Hz, and amplitude of acceleration is always kept at 10 m/s^2^. In order to ensure repeatability of results, the experiment has been repeated three times. The Figure 5 shows a time domain signal of each FBG under the *x*-direction excitation with the acceleration—10 m/s^2^-200 Hz. Figure 5 shows that the sine wave response of #1FBG and #2FBG is very clearly compared with the #3FBG and #4FBG, which is consistent with the real situation. Using Equation (15) to dispose the four FBG time domains, the triaxial vibration curves will be attained in Figure 6. From Figure 6, we can find that: in the *x*-vibration direction, the response is very clear. In addition, there is very little response in the other two directions, which can be ignored compared with response in *x*-vibration direction. Thus, the vibration of *x*-direction has been discerned by the decoupling principle.

**Figure 7:**
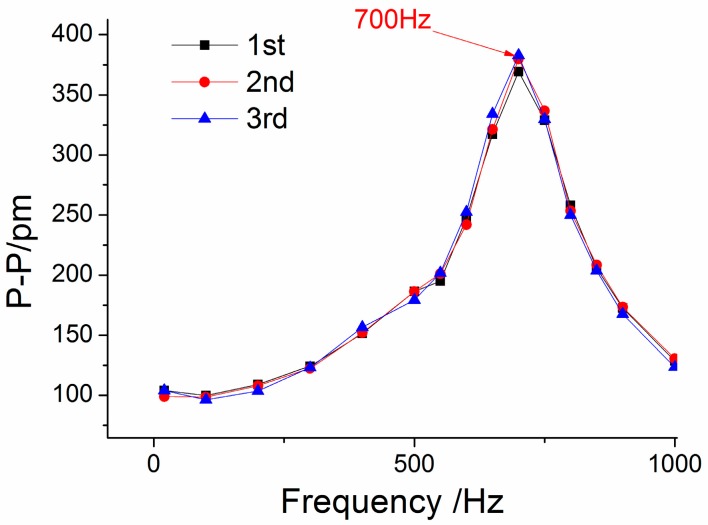
Amplitude-frequency curve of the sensor for the *x*-vibration direction.

**Figure 8:**
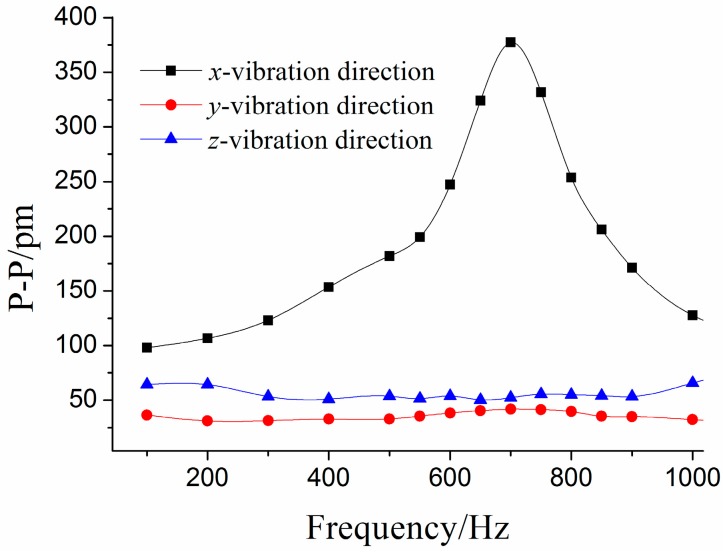
Amplitude-frequency curves of each direction under *x*-direction excitation.

Using a method similar to the above (Figure 6a) to handle the three experiments’ data, we can get the amplitude-frequency curve of the sensor in the *x*-vibration direction (Figure 7). From Figure 7, we can get that when frequency is (i) within 20 to 250 Hz, the curve is almost parallel to the horizontal axis in the *x*-vibration direction; (ii) equal to 700 Hz, the value of P-P reaches the maximum. Based on the above analysis, for the *x*-vibration direction, the working band and the resonant frequency of the sensor are about 10–250 Hz and 700 Hz, respectively. Figure 8 shows the average of center wavelength shifts obtained in the repeated experiments. From Figure 8, we can get that amplitude-frequency curves of the *y/z* direction are almost parallel to horizontal axis, in the *y/z* vibration direction the P-P (Peak-peak) values of curve are separately 32.9 pm and 60.6 pm.

**Figure 9:**
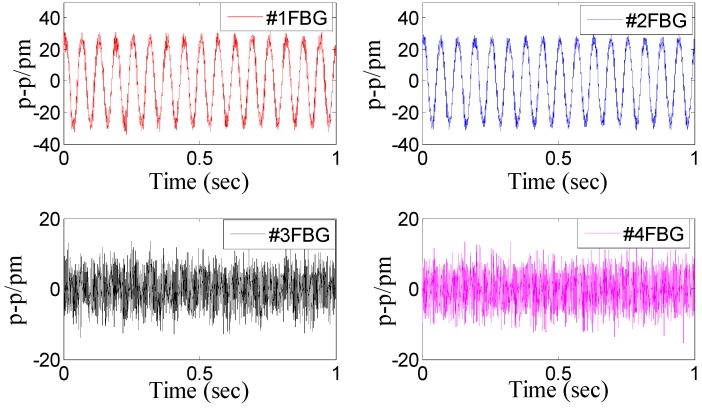
Time domain signal of each FBG under *y*-direction excitation with the acceleration—1m/s^2^-16Hz.

**Figure 10:**
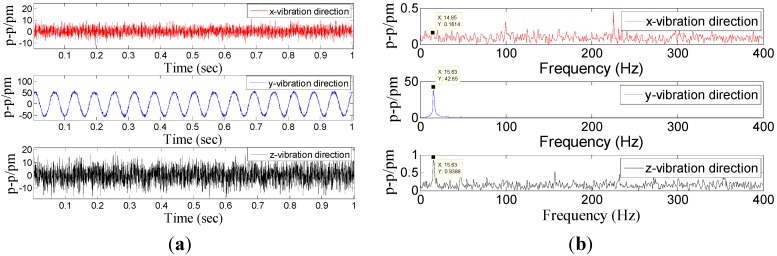
Response of the three directions under *y*-direction excitation with the acceleration— 1m/s^2^-16Hz. (**a**) Time domain response map. (**b**) Spectrum map.

**Figure 11:**
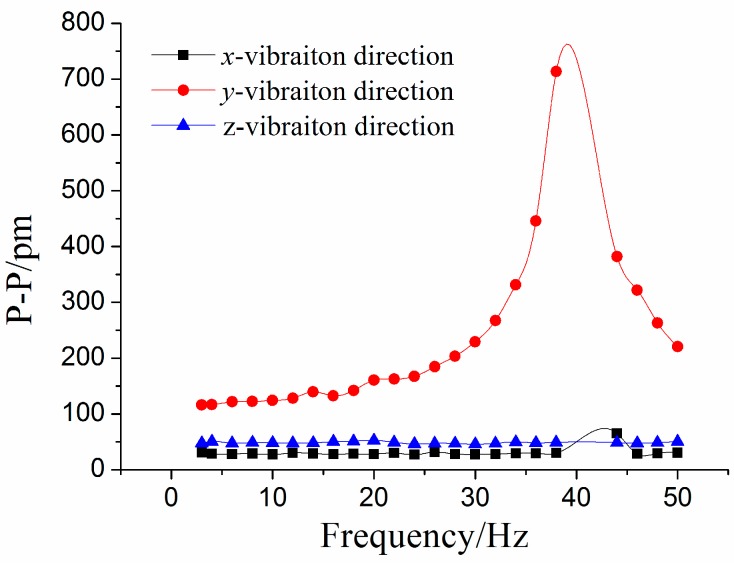
Amplitude-frequency curves of each direction under *y*-direction excitation.

With the purpose of investigating the sensor’s properties in the *y*-main vibration direction, we change the *y*-direction as the main vibration direction. The amplitude of acceleration stays at 1 m/s^2^, and frequency of acceleration increases from 3 to 50 Hz. Figure 9 and Figure 10 are separately showing the response of four FBGs and three direction vibration under *y*-vibration excitation. From the two maps, we can find that the response of the *y*-direction is more clear than the other two directions. After average value processing for experimental data, amplitude-frequency curves of each direction under *y*-direction excitation is shown in Figure 11. From the Figure 11, we can get that when frequency is (i) within 3 to 20 Hz, the curve is almost parallel to horizontal axis in the *y*-vibration direction; (ii) equal to 40 Hz, the value of P-P reaches the maximum. Therefore, for the *y*-vibration direction, the working band and the resonant frequency of the sensor are about 3–20 Hz and 40 Hz, respectively; (iii) the amplitude-frequency curves in the *x/z* vibration direction are almost parallel to horizontal axis, in the *x/z* direction the P-P values of curve are separately 28.7 pm and 48.5 pm.

**Figure 12:**
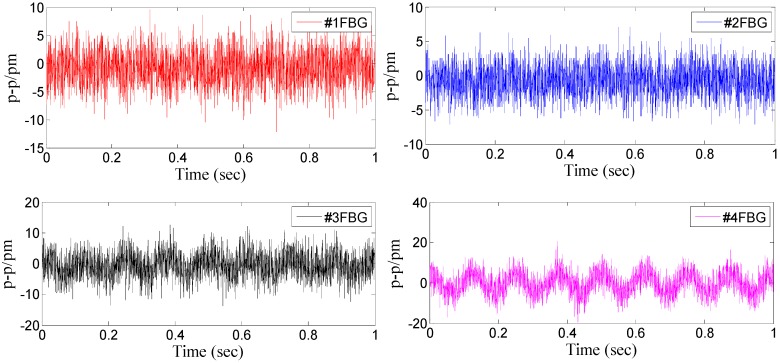
Time domain signal of each FBG under *z*-direction excitation with the acceleration—1 m/s^2^-8 Hz.

**Figure 13:**
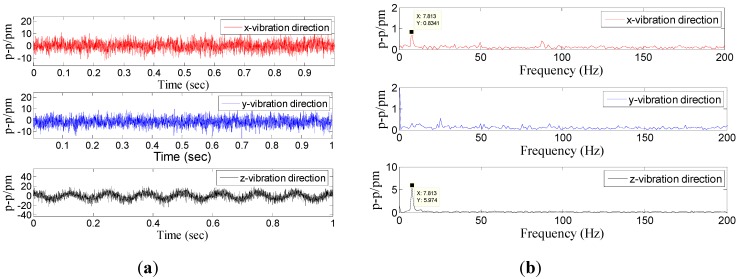
Response of the three directions under *z*-direction excitation with the acceleration—1 m/s^2^-8 Hz. (**a**) Time domain response map. (**b**) Spectrum map.

**Figure 14:**
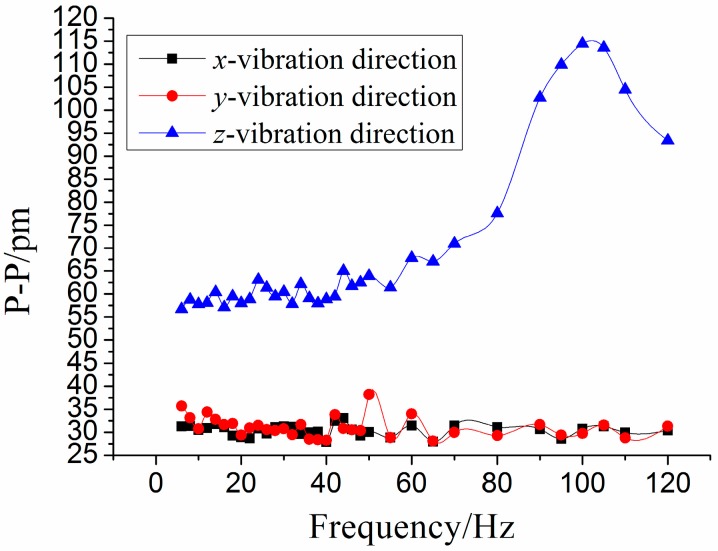
Amplitude-frequency curves of each direction under *z*-direction excitation.

In order to investigate the sensor’s properties in the *z*-main vibration direction, we change the *z*-direction as the main vibration direction. The amplitude of acceleration stays at 1 m/s^2^, and frequency of acceleration increases from 3 to 120 Hz. The response of four FBGs and three direction vibration under *z*-vibration excitation are shown in Figure 12 and Figure 13, respectively. Figure 14 shows the amplitude-frequency curves of each direction under *z*-direction excitation. From Figure 14, we can obtain that when frequency is (i) within 3 to 50 Hz, the curve is almost parallel to horizontal axis for the *z*-vibration direction; (ii) equal to 110 Hz, the value of P-P reaches the maximum. Therefore, for the *z*-vibration direction, the working band and the resonant frequency of the sensor are about 3–50 Hz and 110 Hz, respectively; (iii) the amplitude-frequency curves in the *x/y* vibration direction are almost parallel to horizontal axis, in the *x/y* direction, the P-P values of curve are separately 30.5 pm and 31.6 pm.

### 3.2. Static Characteristic Experiments of the Sensor

**Figure 15:**
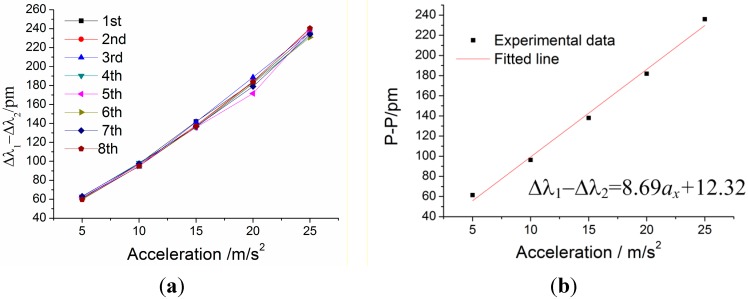
Difference value Δ*λ*_1_ − Δ*λ*_2_ versus acceleration *a_x_* under *x*-direction excitation with the acceleration frequency of 100 Hz. (**a**) Relation between Δ*λ*_1_ − Δ*λ*_2_ and acceleration *a_x_* within 5–25 m/s^2^. (**b**) The linear fitting curve.

In order to research the static properties of the sensor in the *x*-vibration direction, the amplitude of incentive acceleration changes within 5 m/s^2^ to 25 m/s^2^, and frequency of acceleration is always kept at 100 Hz, which is within the working band 10–200 Hz. In order to demonstrate repeatability of the sensor, the experiment has been repeated four times. Figure 15 shows difference value Δ*λ*_1_ − Δ*λ*_2_ versus acceleration *a_x_* under *x*-direction excitation with the acceleration frequency of 100 Hz. From Figure 15a, we can get the sensor’s repeatability error is 6.19% and hysteresis error is 9.80%. In Figure 15b, experimental data is the average of the difference value Δ*λ*_1_ − Δ*λ*_2_ of the repeated experiments. According to the straight line in Figure 15b, we can obtain the following data: (i) sensitivity of the sensor: 86.9 pm/g; (ii) linearity: 3.64%; (iii) fitted equation: Δ*λ*_1_ − Δ*λ*_2_ = 8.69 × *a_x_* + 12.32.

**Figure 16:**
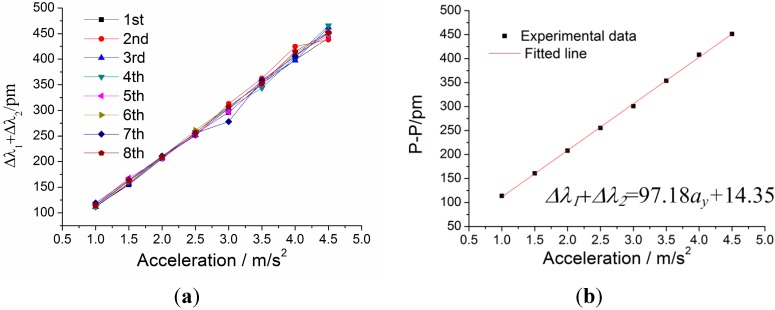
Addition value Δ*λ*_1_ + Δ*λ*_2_ versus acceleration *a_y_* under *y*-direction excitation with the acceleration frequency of 8 Hz. (**a**) Relation between Δ*λ*_1_ + Δ*λ*_2_ and acceleration *a_y_* within 1–4.5 m/s^2^. (**b**) The linear fitting curve.

For the static properties of sensor in the *y*-vibration direction, adjusting the excitation direction along the *y*-vibration direction of sensor. In addition, the amplitude of incentive acceleration changes within 1 m/s^2^ to 4.5 m/s^2^, and frequency of acceleration is always kept at 8 Hz, which is within the working band 3–20 Hz. The experiment is repeated four times. Figure 16 shows addition value Δ*λ*_1_ + Δ*λ*_2_ versus acceleration *a_y_* under *y*-direction excitation with the acceleration frequency of 100 Hz. From Figure 16a, we can get that the sensor’s repeatability error is 8.65% and hysteresis error is 8.15%. In Figure 16b, experimental data is the average of the difference value Δ*λ*_1_Δ*λ*_2_ in the repeated experiments. According to the straight line in Figure 16b, we can obtain the following data: (i) sensitivity of the sensor: 971.8 pm/g; (ii) linearity: 1.50%; (iii) fitted equation: Δ*λ*_1_ + Δ*λ*_2_ = 97.18 × *a_y_* + 14.35.

**Figure 17:**
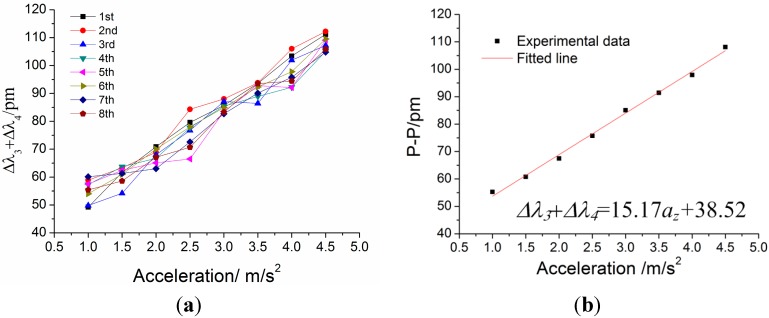
Addition value Δ*λ*_3_ + Δ*λ*_4_ versus acceleration *a_z_* under *z*-direction excitation with the acceleration frequency of 8 Hz. (**a**) Relation between Δλ_3_ + Δλ_4_ and acceleration *a_z_* within 1–4.5 m/s^2^. (**b**) The linear fitting curve.

For the static properties of sensor in the *z*-vibration direction, the amplitude of incentive acceleration changes within 1 m/s^2^ to 4.5 m/s^2^, and frequency of acceleration is always kept at 8 Hz, which is within the working band 3–50 Hz. The experiment is repeated four times. Figure 17 shows addition value Δ*λ*_3_ + Δ*λ*_4_ versus acceleration *a_z_* under *z*-direction excitation with the acceleration frequency of 100 Hz. According to the straight line in Figure 17b, we can obtain the following data: (i) sensitivity of the sensor: 151.7 pm/g; (ii) linearity: 3.01%; (iii) fitted equation: Δ*λ*_3_ + Δ*λ*_4_ = 15.17 × *a_z_* + 38.52.

### 3.3. Discussions and Analysis

**Figure 18:**
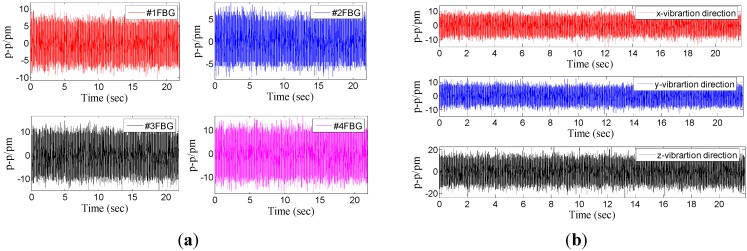
Response of each FBG and three directions without excitation. (**a**) Time domain signal of each FBG without excitation. (**b**) Response of the three directions without excitation.

From the above analysis of the sensor’s dynamic/static characteristics, we can find that: (i) according to Equation (15), the decoupling triaxial vibration can be easily attained by the center wavelength shift of four FBGs. Thus, the FBG based triaxial vibration sensor can be used to measure the three-dimensional vibration. (ii) When the triaxial vibration sensor vibrates in a single direction, the other two directions also display certain outputs. This phenomenon is mainly caused by the installation error, the precision of FBG interrogators, and the nonlinear factors of the sensor model. In order to further explain this problem and analyze the interference characteristics of the triaxial sensor in each direction, we have extracted the response of each FBG and three directions without excitation (Figure 18). The maximum of response is extracted in Table 2. From Table 2, we can get that: when the triaxial vibration sensor is used to measure a single direction vibration, the outputs of the other two directions are almost equal to the results without excitation. It is further proved that this method can be used to decouple the triaxial vibration.

**Table 2:** Response of fiber Bragg grating (FBG) based triaxial sensor under different excitation.

(*a_x_*, *a_y_*, *a_z_*)/m/s^2^	Δ*λ*_1_ − Δ*λ*_2_/pm	Δ*λ*_1_ + Δ*λ*_2_/pm	Δ*λ*_3_ + Δ*λ*_4_/pm
(0,0,0)	29.2	28.7	46.5
(10,0,0)	109	32.9	60.6
(0,1,0)	28.7	123	48.5
(0,0,1)	30.5	31.6	59.1

## 4. Conclusions

A fiber Bragg grating sensing based triaxial vibration sensor has been presented in this paper. The optical fiber is directly employed as an elastomer, and the triaxial vibration of measured body can be obtained by two pairs of FBGs. A model of a triaxial vibration sensor as well as decoupling principles of triaxial vibrations and experimental analyses are proposed. Experimental results show that: (1) Sensitivities of 86.9 pm/g, 971.8 pm/g and 154.7 pm/g for each orthogonal sensitive direction with linearity are separately 3.64%, 1.50% and 3.01%. (2) Fitted equations of each direction: Δ*λ*_1_ − Δ*λ*_2_ = 8.69 × *a_x_* + 12.32, Δ*λ*_1_ + Δ*λ*_2_ = 97.18 × *a_y_* + 14.35 and Δ*λ*_3_ + Δ*λ*_4_ = 15.17 × *a_z_* + 38.52. (3) The flat frequency ranges reside in 20–200 Hz, 3–20 Hz and 4–50 Hz, respectively. (4) In addition, the resonant frequencies are separately 700 Hz, 40 Hz and 110 Hz in the *x*/*y*/*z* direction. When the sensor is excited in a single direction vibration, the outputs of the sensor in the other two directions are consistent with the outputs in the non-working state. It is effectively demonstrated that it can be used to decouple the triaxial vibration and measure three-dimensional vibration.
